# Supplementary material for: A New Strategy for Nucleic Acid Delivery and Protein Expression Using Biocompatible Nanohydrogels of Predefined Sizes
Source: Pharmaceutics. 2023 Mar 16;15(3):961. doi: 10.3390/pharmaceutics15030961 (PMC10058534; doi:10.3390/pharmaceutics15030961)
Supplement: Supplementary file 1 [file pharmaceutics-15-00961-s001.zip › pharmaceutics-2185681-supplementary.pdf]

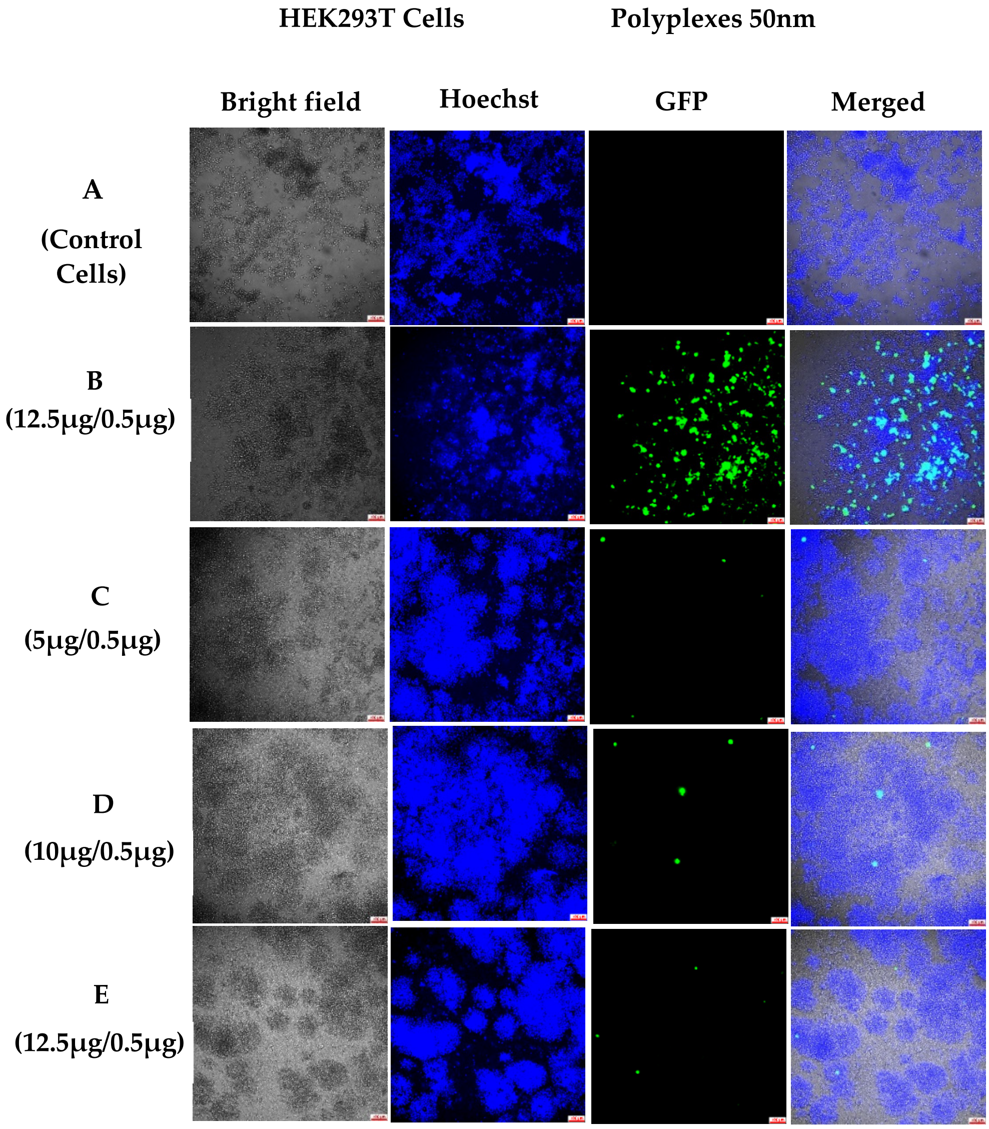

Figure S1. Fluorescence microscopic images (10X) of 50 nm polyplexes with HEK293T cells after 6 days incubation. (Scale bar for all the pictures - 100μm). (A) Control cells, (B) 12.5 μg NHG's (200nm)/0.5 μg GFP, (C) 5 μg NHG's (50nm)/0.5 μg GFP, (D) 10 μg NHG's (50nm)/0.5 μg GFP and (E) 12.5 μg NHG's (50nm)/ 0.5 μg GFP.

.

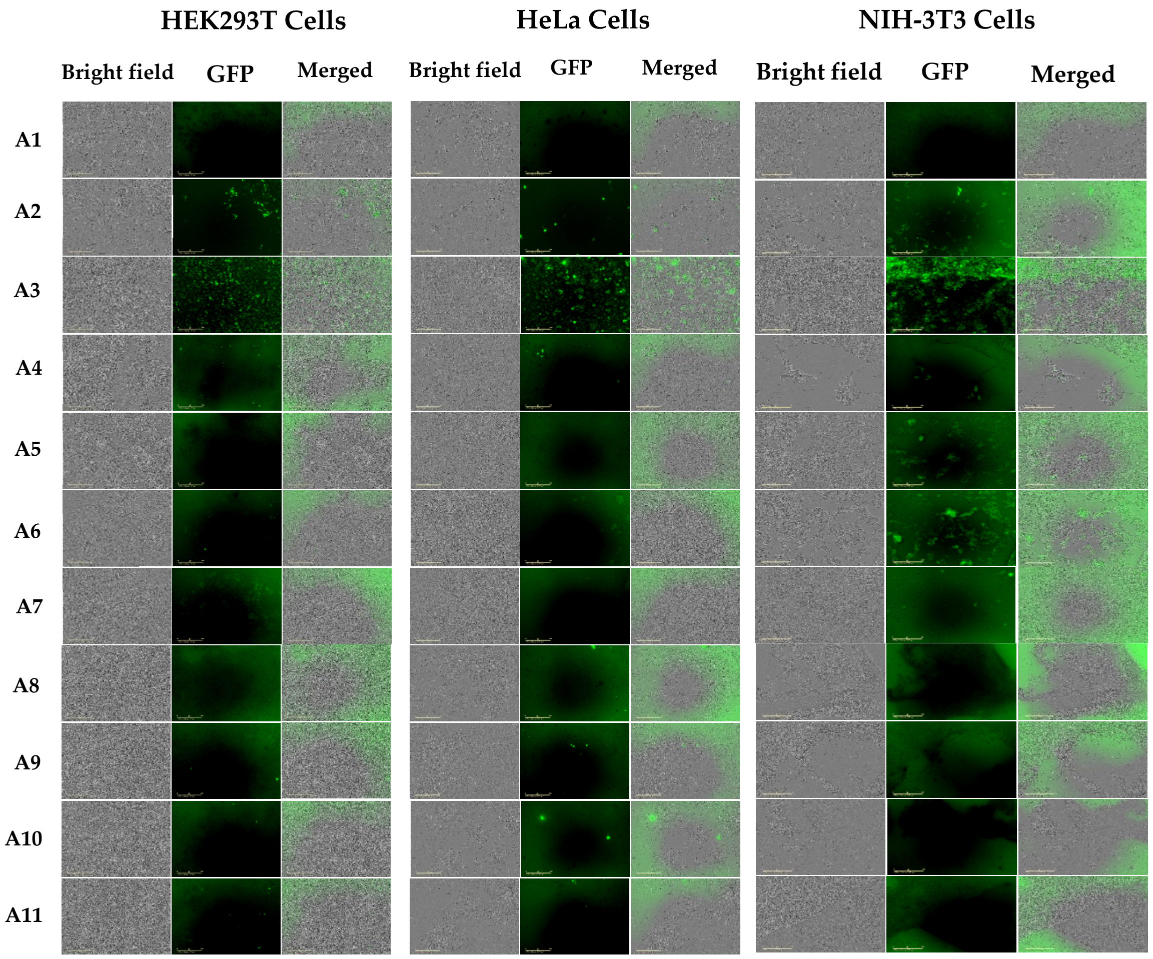

Figure S2. Imaging with Incucyte system (10X) for polyplexes (400, 200 and 50nm) with HEK293T, HELA and NIH-3T3 cells after 1 week incubation (scale bar 400µm, area: 1.75 x 1.29 mm, 2.27mm2). (A1) Control cells, (A2) 6nmol lipid/ 0.5µg GFP, (A3) 12.5µg NHG's/ 0.5µg GFP (200nm), (A4) 2.5µg NHG's/0.5µg GFP (400nm), (A5) 5µg NHG's/0.5µg GFP (400nm), (A6) 10µg NHG's/0.5µg GFP (400nm), (A7) 12.5µg NHG's/ 0.5µg GFP (400nm), (A8) 10µg NHG's/0.5µg GFP (50nm), (A9) 12.5 µg NHG's/0.5µg GFP (50nm) (A10) 15µg NHG's/0.5µg GFP (50nm) and (A11) 20µg NHG's /0.5µg GFP (50nm).

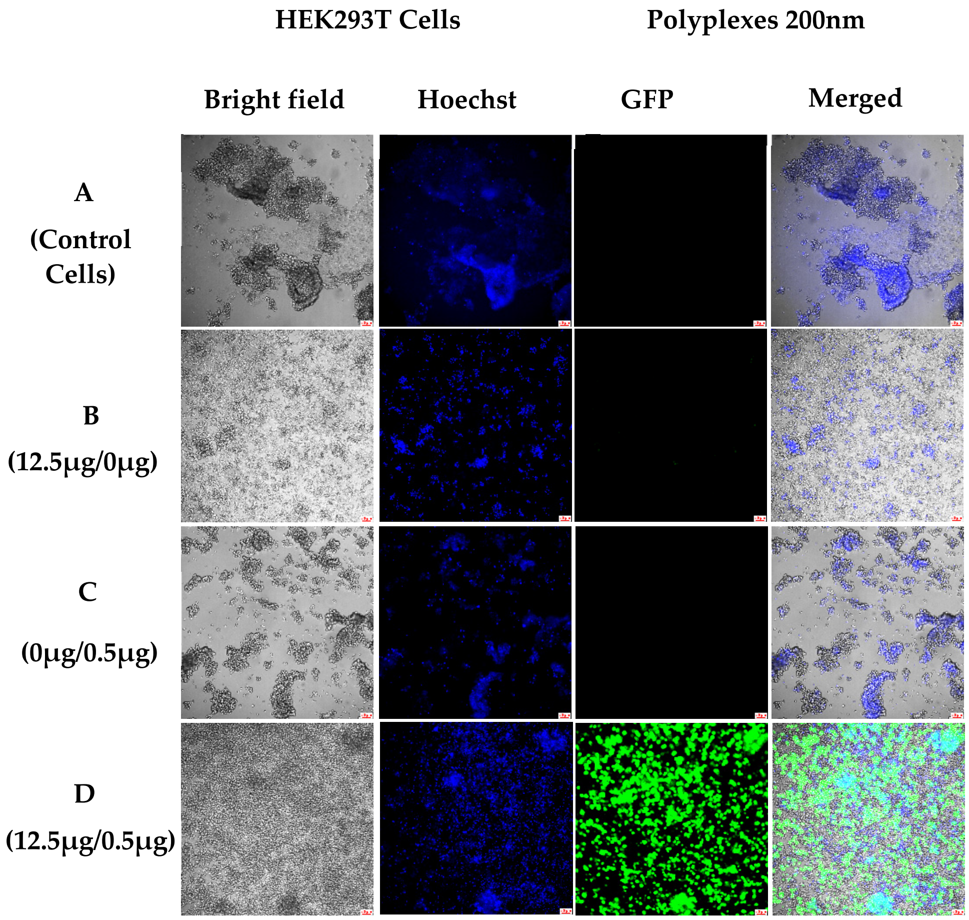

Figure S3. Fluorescence microscopic images (10X) of 200 nm polyplexes with HEK293T cells after 6 days incubation (10% FBS from time zero) (Scale bar for all the pictures - 100µm). (A) Control cells, (B) 12.5 µg NHG's alone, (C) 0.5 µg GFP alone, and (D) 12.5 µg NHG's/0.5 µg G

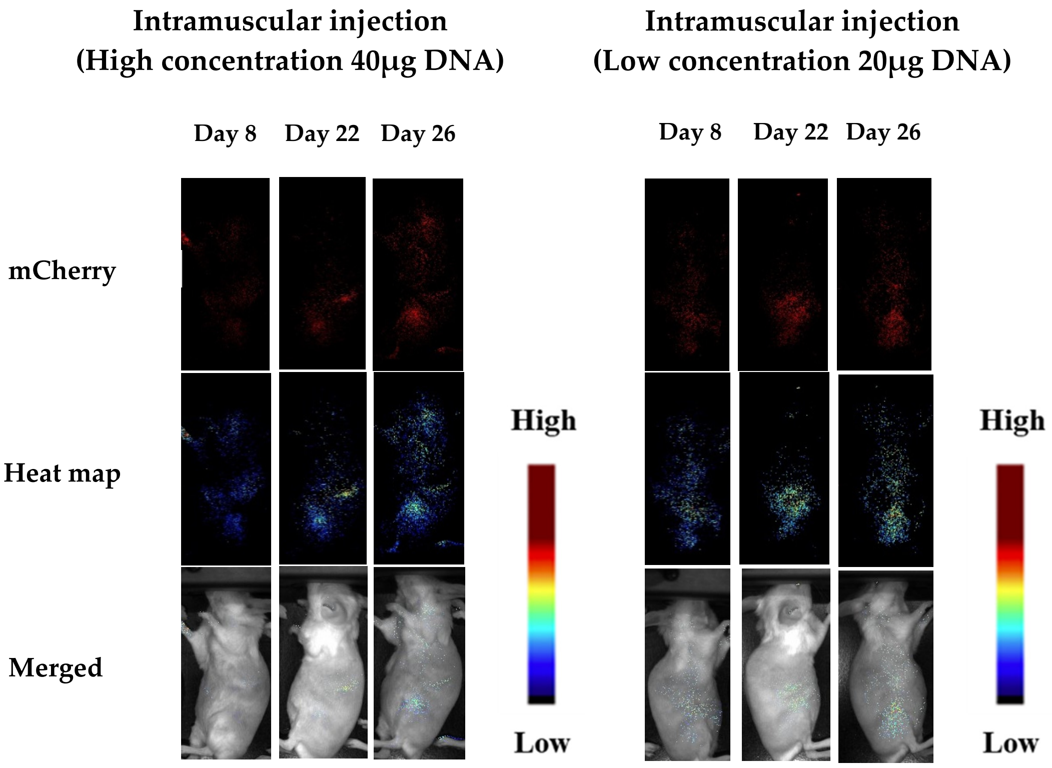

Figure S4. Detection of m-Cherry after Intramuscular administration for days 8, 22 and 26.

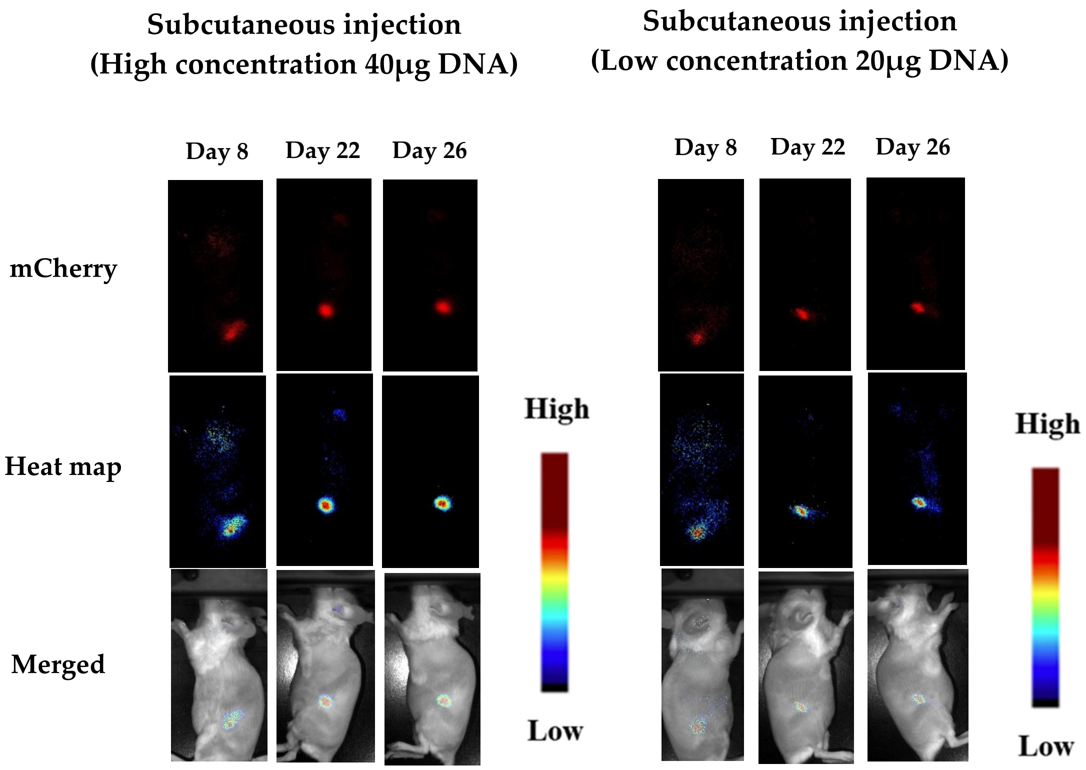

Figure S5. Detection of m-Cherry after subcutaneous administration for days 8, 22 and 26.
